# Synthetic and Bio-Derived Surfactants Versus Microbial Biosurfactants in the Cosmetic Industry: An Overview

**DOI:** 10.3390/ijms22052371

**Published:** 2021-02-27

**Authors:** Ana B. Moldes, Lorena Rodríguez-López, Myriam Rincón-Fontán, Alejandro López-Prieto, Xanel Vecino, José M. Cruz

**Affiliations:** 1 Chemical Engineering Department, School of Industrial Engineering—Cintecx, Campus As Lagoas-Marcosende, University of Vigo, 36310 Vigo, Spain; lorena@uvigo.es (L.R.-L.); myriam@uvigo.es (M.R.-F.); alexlopez@uvigo.es (A.L.-P.); jmcruz@uvigo.es (J.M.C.); 2 Chemical Engineering Department, Barcelona East School of Engineering (EEBE)—Barcelona Research Center for Multiscale Science and Engineering, Campus Diagonal-Besòs, Polytechnic University of Catalonia (UPC), 08930 Barcelona, Spain

**Keywords:** petroleum-based surfactants, bio-based surfactants, microbial surfactants, cosmetics, industrial application

## Abstract

This article includes an updated review of the classification, uses and side effects of surfactants for their application in the cosmetic, personal care and pharmaceutical industries. Based on their origin and composition, surfactants can be divided into three different categories: (i) synthetic surfactants; (ii) bio-based surfactants; and (iii) microbial biosurfactants. The first group is the most widespread and cost-effective. It is composed of surfactants, which are synthetically produced, using non-renewable sources, with a final structure that is different from the natural components of living cells. The second category comprises surfactants of intermediate biocompatibility, usually produced by chemical synthesis but integrating fats, sugars or amino acids obtained from renewable sources into their structure. Finally, the third group of surfactants, designated as microbial biosurfactants, are considered the most biocompatible and eco-friendly, as they are produced by living cells, mostly bacteria and yeasts, without the intermediation of organic synthesis. Based on the information included in this review it would be interesting for cosmetic, personal care and pharmaceutical industries to consider microbial biosurfactants as a group apart from surfactants, needing specific regulations, as they are less toxic and more biocompatible than chemical surfactants having formulations that are more biocompatible and greener.

## 1. Introduction

Surfactants are amphiphilic compounds that contain a tail, which is insoluble in water, presenting hydrophobic groups and a water-soluble head with hydrophilic groups [1,2]. Due to this structure, these substances have the ability to diffuse in water and to place themselves between air/water or oil/water interfaces [3,4], solubilizing hydrophobic compounds in water and giving stable emulsions with many applications in different industrial sectors including the agrochemical [5,6,7], agriculture [8], food [9,10,11], pharmaceutical [12] and cosmetic industries [13,14] as well as therapeutic [15], medicine [16] and oral-health related [17] applications.

The synthesis of surfactants usually involves chemical reactions to combine a hydrophobic chain with a hydrophilic group. Surfactants can be classified from different points of view [2], as there is currently no official categorization. Usually, they are classified depending on their ionic charge, although this classification can only be applied to synthetic and bio-based surfactants since the charge of most surfactants produced by living cells is still unknown. At the moment, only a few researchers have paid attention to the ionic charge or to the hydrophilic–lipophilic balance (HLB) of microbial biosurfactants [18]. Both parameters, together with the critical micellar concentration (CMC), are the important features of microbial biosurfactants and also important properties for cosmetic applications [19,20]. For instance, skin and eye irritation, absorption by hair and antibacterial properties can be associated with the ionic charge of microbial biosurfactants [21,22]; while the HLB value influences the ability of microbial biosurfactants to act as wetting, anti-foaming or emulsifier agents, among others purposes [14,22].

Other classifications divide surfactants into synthetic surfactants and bio-based surfactants, including also as bio-based surfactants produced by living cells, called microbial biosurfactants. Bio-based surfactants are usually produced by chemical synthesis but integrating fats, sugars or amino acids obtained from renewable sources, whereas microbial surfactants are obtained from living cells, typically bacteria and yeasts, without the intermediation of organic synthesis. However, the synthesis and composition of bio-based surfactants produced by organic synthesis differ from the surfactants directly produced from living cells and, therefore, they should be classified separately [2,23].

Microbial biosurfactants are secondary metabolites produced by different microorganisms, including bacteria, fungus and yeast [24]. They can be associated with different stages of microbial growth [25] and most of them are produced extracellularly, although there are some microorganisms that are able to produce microbial biosurfactants linked to the plasmatic membrane, called cell-bound microbial biosurfactants. Usually, lipopeptides and glycolipids compose extracellular microbial biosurfactants [26,27,28,29,30], whereas cell-bound microbial biosurfactants are composed of glycolipids and glycolipopeptides [31,32,33,34,35].

Another group of substances with surfactant capacity that can be considered biosurfactants, as they are extracted from vegetable or animal cells, are phospholipids [36]. However, it is important to remark that the main function of phospholipids at an industrial scale is emulsifiers. Moreover, some microorganisms also produce polymeric and particulate substances (named viscosin or emulsan) with surfactant capacity, although their main property is also as an emulsifier [24]. These groups of substances have similar properties to phospholipids and, therefore, they should be included in the same group. The aim of this review is based on the study of similarities and differences in the classification, uses and side effects between synthetic and bio-based surfactants in comparison with microbial biosurfactants for cosmetic, personal care and pharmaceutical applications.

## 2. Synthetic Surfactants

Synthetic surfactants are usually classified on the basis of their ionic charge as cationic, anionic, non-ionic and zwitterionic or amphoteric surfactants [2]. The most common anionic surfactants are composed of sulfate, sulfonate, phosphate and carboxylate salts. For instance, within this category, it is possible to find organosulfates like sodium lauryl sulfate (SLS), which is a mixture of sodium alkyl sulfates where the main component is sodium dodecyl sulfate (SDS); SDS and related potassium and ammonium salts. The most used cationic surfactants include protonated primary, secondary and tertiary amines as well as quaternary ammonium salts, the most used being cetrimonium bromide (CTAB), cetylpyridinium chloride (CPC), benzalkonium chloride (BAC), benzethonium chloride (BZT), dimethyldioctadecylammonium chloride (DODMAC), and dioctadecyldimethylammonium bromide (DODAB) [2,37]. On the other hand, zwitterionic surfactants are tensides that contain both positive and negative charges. These charges can either be permanent or dependent on the pH value of the medium. Frequently, the cationic moiety is either an amine or a quaternary ammonium cation, whereas the anionic part is mostly a carboxylate, sulfonate, sulfate or phosphate group. The latter group, zwitterionic or amphoteric surfactants, includes carboxylic acids, quaternary ammonium ions, aromatic quaternary ammonium surfactants and betaine derivatives [2].

Zwitterionic surfactants are, among synthetic surfactants, those that can compete with microbial biosurfactants, as they are considered more biocompatible and have fewer side effects than anionic or cationic surfactants. An example of zwitterionic surfactants is the combination of a carboxylic acid and quaternary ammonium ions, such as amidosulfobetaine-16, which has a longer hydrophobic tail, or the C80 detergent, which has an alkylphenyl hydrophobic tail; other carboxylic acids/quaternary ammonium surfactants include lauryl-*N,N*-(dimethylammonio)-butyrate and lauryl-*N*,*N*-(dimethyl)-glycinebetaine [38].

Some zwitterionic surfactants are obtained by combination of sulfuric acid and a quaternary ammonium ion. Surfactants of this type include the hydroxysultaines class such as 3-[(3-cholamidopropyl)dimethylammonio]-1-propanesulfonate (CHAPS). A slightly modified version is 3-[(3-cholamidopropyl)dimethylammonio]-2-hydroxy-1-propanesulfonate, which is slightly more hydrophilic than CHAPS. There are also several aromatic quaternary ammonium surfactants which possess zwitterionic properties, as is the case of 3-(4-tert-butyl-1-pyridinio)-1-propanesulfonate, 3-(1-pyridinio)-1-propanesulfonate, and 3-(benzyl-dimethylammonio)propanesulfonate [38].

Additionally, zwitterionic surfactants can be obtained by reaction of phosphoric acid and quaternary ammonium ions, as is the case of the other acids mentioned above. Another group of zwitterionic surfactants is obtained from quaternary ammonium ions with an attached hydroxy group. A typical example of this class of surfactants is lauryldimethylamine *N*-oxide [38].

Finally, this group of surfactants includes betaines, which can exist in two ionic forms, zwitterionic and cationic [39]. Zhou et al. [40] synthetized a novel oligomer betaine surfactant by substitution, quaternization and neutralization reactions: penta sodium *N*, *N′*, *N″*- dodecyl diethylene triamine pentaacetic acid (DDTPA) with three dodecyls, five carboxylic acid groups and three quaternary ammoniums. The above-mentioned betaine synthetized possesses better surface-active properties than the conventional betaine derivatives.

## 3. Bio-Based Surfactants

Bio-based surfactants can also be classified as anionic, non-ionic, cationic and amphoteric surfactants. Below, different examples of each group are presented [23].

### 3.1. Anionic

#### 3.1.1. Methyl Ester Sulfonates (MES)

Fatty acid methyl ester sulfonates (MES) are oleochemical-based anionic surfactants, derived mainly from palm or coconut oil, obtained by a sulfonation reaction followed by acid digestion. MES, linear alkylbenzene sulfonates (LASs), alkyl sulfonates (ASs), and alpha olefin sulfonates (AOSs), are used as detergent surfactants [41]. MES comprises the sodium salts of the sulfonated methyl ester acids, such as lauric acid (sodium methyl laurate sulfonate), myristic acid (sodium methyl myristate sulfonate) palmitic acid (sodium methyl palmitate sulfonate) and stearic acid (sodium methyl stearate sulfonate).

LASs are fossil fuel-derived surfactants that can possess good bio-degradability and low ecotoxicity. However, due to the interest of detergent companies in natural fat- and oil-based surfactants, rather than those that are petroleum-based like LAS, MES are considered as a main laundry detergent ingredient with good surface-active properties as well as good biodegradability [23,41,42,43].

This type of bio-based surfactant has been defined by some authors as a high-performance surfactant, capable of reducing builders dosage in detergents, presenting a superior soil removal index and detergency, in comparison to alkyl benzene sulfonates [44]. Moreover, they possess liquid crystal structures, which are essential for the stabilization of foams and emulsions in lubrication and other applications [45].

#### 3.1.2. Alcohol Sulfates (AS) and Alcohol Ether Sulfates (AES)

This group includes C10–C18 alcohol sulfates containing, for instance, coconut fatty alcohol sulfates or from ethylene via Ziegler C12/14 alcohol. Depending on the length of the fatty acid chain, detergents with different properties can be obtained. Those containing short fatty acids (C12–C14) are used in a wide range of personal care products like shampoos, bubble bath products, toothpaste, dishwashing liquids and products for delicate laundry washing [46].

In addition, ammonia or amine salts of lauryl alcohol sulfates are also used in shampoos and bubble products, whereas sodium salts of C16–C18 alcohol sulfates are used in the formulation of heavy-duty laundry products for hand and machine washing, with a detergency power higher than ammonium lauryl sulfate (ALS) [46].

In general, alcohol ether sulfates (AES) are considered the most efficient in terms of detergency, good tolerance to hard water and mildness; however, they are very unstable at high temperatures [46]. This group includes Sodium Lauryl Sulfate (SLS), Sodium Coco Sulfate (SCS) and Sodium Laureth Sulfate (SLES) [47,48]. All three are obtained from coconut oil, but there are differences between them. To obtain SCS, pure coconut oil (with all its fatty acids) is sulfated by reaction with sulfur trioxide, followed by neutralisation (usually with NaOH). This results in a detergent that does not produce much foam, although this may vary depending on the quality of the coconut crop in question [47].

SLS is a purified version of the SCS. In this case, most of the non-carbon 12 fatty acids in coconut oil are removed. The starting material is then 80% carbon 12 fatty acids (mainly lauric acid) subjected to the same sulfation process as in the previous case.

Both products, SCS and SLS, are mainly sodium lauryl sulfate (because in both cases lauric alcohol predominates), with SLS being more efficient, and SCS a highly diluted, and therefore milder form [47].

Detergents companies use SLS in some products and SLES in others, depending on the purpose of the product. However, for example, SLS can be irritating to the skin so many manufacturers use SLES instead. SLES is prepared similarly to SLS, but by going through a process of ethoxylation of dodecyl alcohol (lauric alcohol) and then sulfated. The addition of ethylene oxide to the fatty alcohols in the formula makes them more soluble in water, which reduces the level of irritation. Thus, SLES turns out to be a detergent with high skin compatibility, and great moisturizing and emulsifying capacity [48]. However, in the process of ethoxylation of lauric alcohol (derived from lauric acid) with ethylene oxide, an ingredient called 1,4-dioxane can be obtained as a by-product, which is considered carcinogenic [49]. Nevertheless, manufacturers have optimised the process so as to achieve very low levels of this by-product.

#### 3.1.3. Glycinate Amino Acid-Based Surfactants

These bio-based surfactants can be synthesized using cocoyl chloride, derived from fatty acid, and phosphorus trichloride. Recently, some authors have proposed the use of coconut oil in combination with amino acids in order to obtain more eco-friendly bio-based glycinate surfactants [50]. Examples of these bio-based surfactants are potassium cocoyl glycinate and sodium cocoyl glycinate. Regarding its production, potassium cocoyl glycinate is prepared by setting the temperature reaction at 60 °C in the presence of coconut oil and phosphorus trichloride. As by-products, phosphorus acid and glycerine are obtained. For sodium cocoyl glycinate, however, the reaction is carried out in the presence of glycine, KOH and cocoyl chloride, at pH 11.5–12.5 [50,51].

### 3.2. Cationic

#### Glycine Betaine Esters and Amides

This group includes a specific type of cationic surfactant based on natural glycine betaines and vegetable oils, produced from renewable raw materials. Glycine betaine is a natural low-expense substance, which possesses a quaternary trimethylalkylammonium moiety and a carboxylate function. It constitutes the main raw material for the preparation of biodegradable and biocompatible cationic surfactants such as glycine betaine esters and amides. It accounts for 27% of the weight of molasses of sugar beet (*Beta vulgaris*) and is obtained after extraction of saccharose. This compound reacts with electrophilic reagents such as alkyl halides and their tosylate counterparts to obtain the ester-type surfactant. Conversely, glycine betaine reacts with n-alcohol and fatty amines such as lauric, stearic and oleic amines, in order to obtain the amides [52].

### 3.3. Non-Ionic

#### Sugar Bio-Based Surfactants

There are several types of sugar surfactants such as sucrose esters, methyl glycoside esters, ethyl glycoside esters, *N*-methyl-glucamides or sorbitan esters, with alkyl polyglucosides (APGs) being the most important group. These surfactants are composed of a fatty acid tail, which contains from 8 to 14 carbon atoms, and a sugar moiety composed of glucose, sucrose, galactose, mannose and other sugars. They are composed of sugars derived from plants, which makes them biodegradable [53]. When the fatty acids of APGs are derived from coconut oil, they are called coco glucosides, and are typically included in many cosmetic formulations. This class of non-ionic surfactants is widely used in cosmetic, household and other industrial applications. Usually, bio-based surfactants like these are produced by combining sugars such as glucose, with fatty alcohol in the presence of acids, the reaction being catalyzed by elevated temperatures [54,55]. There are several types of sugar surfactants such as sucrose esters, methyl glycoside esters, ethyl glycoside esters, *N*-methylglucamides or sorbitan esters, with alkyl polyglucosides (APGs) being the most important group. These surfactants are composed of a fatty acid tail, which contains from 8 to 14 carbon atoms, and a sugar moiety composed of glucose, sucrose, galactose, mannose and other sugars. When the fatty acids of APGs are derived from coconut oil, they are called coco glucosides, and are typically included in many cosmetic formulations.

Gaudin et al. [56] described how that the chemical nature of a substance linked to a sugar molecule possesses an important impact on the surfactant properties. For example, surfactants with thioether linkers were found to have a lower CMC and to be less soluble than their analogues with ether linkers. On the other hand, surfactants with methyl amide linkers were revealed to be more soluble in comparison to surfactants with free amide linkers.

The sugar molecule constitutes the hydrophilic head and the fatty acid residue is considered the hydrophobic tail of the molecule. It is possible to obtain both components from renewable sources and secondary raw materials.

Another group of non-ionic bio-based surfactants is the polysorbates formed by the ethoxylation of sorbitan esters. For instance, Polysorbate 20 is a non-ionic surfactant, formed by the ethoxylation of sorbitan before the addition of lauric acid. Its stability and relative non-toxicity allow it to be used as a detergent and emulsifier in a number of domestic, scientific and pharmacological applications. As the name implies, the ethoxylation process leaves the molecule with 20 repeated units of polyethylene glycol; these are distributed across four different chains, leading to a commercial product containing a range of chemical species [57,58].

Conversely, polysaccharide biomass is used to synthesize sorbitan esters such as the non-ionic surfactants Tween and Span (through the single dehydration of sorbitol) and to produce isosorbide, which can be obtained from the starch industry [59]. Isosorbide is described as a rigid bicyclic diol, containing two-bonded furan rings. It is produced from D-sorbitol obtained by catalytic hydrogenation of D-glucose, which in turn can be produced by hydrolysis of starchy or lignocellulose residues. Isosorbide has the property of acting as a rigid hydrophilic linker between aliphatic chains and hydroxyl and hydrophilic heads. Lavergne et al. [60] have coupled a dodecyl alkyl chain and triethyleneglycol or glycerol to isosorbide, in order to provide the non-ionic polar groups to obtain isosorbide non-ionic surfactants, whereas Mouria-Bellabdelli et al. [61] have synthetized a series of isosorbide diesters and cyclodextrins to create host–guest surfactants, observing that only the isosorbide esters with alkyl chains ranging from C10 to C18 form stable inclusion complexes with the native β-cyclodextrin.

In this regard, native cyclodextrins are oligosaccharides composed of six or more D-glucopyranose residues attached by β-1,4-linkages in a cyclic array. The most common cyclodextrins contain six, seven or eight glucose residues and are named α-cyclodextrin, β-cyclodextrin and γ-cyclodextrin, depending on the number of glucose residues, respectively. Machut et al. [62] have also proven that isosorbide dioleate and sorbitan trioleate formed well-defined inclusion complexes with β-cyclodextrin with surfactant properties, which were obtained by non-covalent associations between β-cyclodextrin and highly hydrophobic polyesters containing several alkyl chains.

Recently, Cho et al. [63] have proposed a new method for the synthesis of isosorbide-based sulfonate anionic surfactants, with hexyl, octyl, decyl, and dodecyl side chains, in the presence of KOH and toluene or using lithium hydroxide and alkyl bromide in dimethyl sulfoxide.

Other authors have proposed the synthesis of derived isosorbide surfactants (dodecylamino diglycidyl ether of isosorbide) by the condensation of isosorbide and epichlorhydrin yielding a low molar mass prepolymer and subsequent condensation to a fatty amine [64]. For instance, isosorbide is commercialized by Roquette company as POLYSORB^®^ isosorbide, which is a cycloaliphatic monomer obtained from sorbitol dehydration that can be used for the synthesis of bio-based isosorbide surfactants.

### 3.4. Amphoteric

#### Cocoamidopropyl Betaine

Cocoamidopropyl is an example of an amphoteric betaine bio-based surfactant produced by the reaction of dimethylaminopropylamine with fatty acids (lauric acid or its methyl ester) from different sources such as coconut or palm kernel oil. In this sense, despite the name, cocamidopropyl betaine is not synthesized from betaine. Moreover, it is considered a mild surfactant, although it can produce some allergic reactions [65,66,67].

On the other hand, the BASF company has started to commercialize an algal betaine surfactant made from renewable microalgae oil as an alternative to cocoamidopropyl.

## 4. Microbial Biosurfactants

Contrarily to chemically synthesized and bio-based surfactants, which are classified according to the nature of their polar group, microbial biosurfactants can be classified based on the chemical nature, on the ionic charge and on the producer microorganism [24], although usually microbial biosurfactants are classified according to their polymeric composition in four main categories: (i) glycolipids; (ii) lipopeptides and lipoproteins; (iii) glycopeptides and glycolipopeptides, and (iv) bioemulsifiers with biosurfactant capacity (phospholipids, polymeric biosurfactants and particulate biosurfactants) [1].

### 4.1. Glycolipids

**Figure 1:**
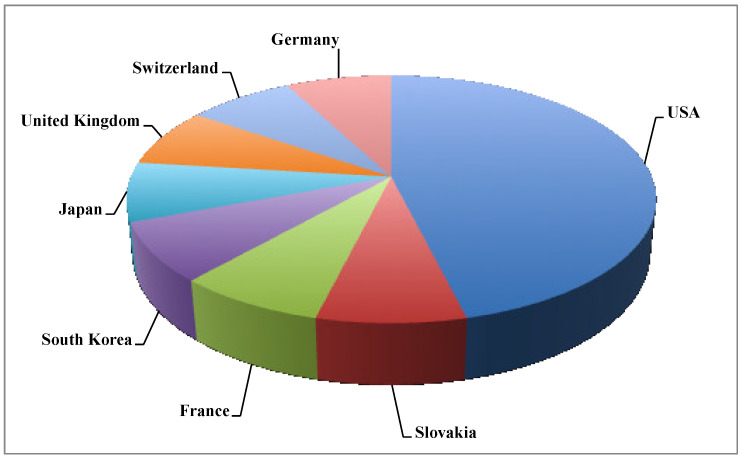
Distribution of global microbial biosurfactant producers by countries based on the number of companies.

Glycolipids are the most common and popular microbial biosurfactants [68] with the largest number of companies dedicated to their production. The most common glycolipids are rhamnolipids and sophorolipids. Sophorolipids are produced by Evonik (Slovenská Ľupča, Slovakia), Groupe Soliance (Pomacle, France), MG Intobio Co. Ltd. (Incheon, South Korea), Synthezyme LLC (Rensselaer, NY, USA) and Saraya Co. Ltd. (Osaka, Japan); whereas rhamnolipids are produced by Logos Technology (Fairfax, VA, USA), TeeGene Biotech (Redcar, United Kingdom), AGAE Technologies LLC (Corvallis, OR, USA), Glycosurf (Park City, UT, USA), Biotensidon GmbH (Zug, Switzerland), Jeneil Biosurfactant Co. LLC (Saukville, WI, USA), Rhamnolipid Companies, Inc. (St. Petersburg, FL, USA) and Henkel (Düsseldorf, Germany). Figure 1 shows the distribution of companies that commercialize microbial biosurfactants, revealing that the market is dominated by the United States. In Europe, only four countries are considered microbial biosurfactants producers: Slovakia, France, Switzerland and Germany.

**Figure 2:**
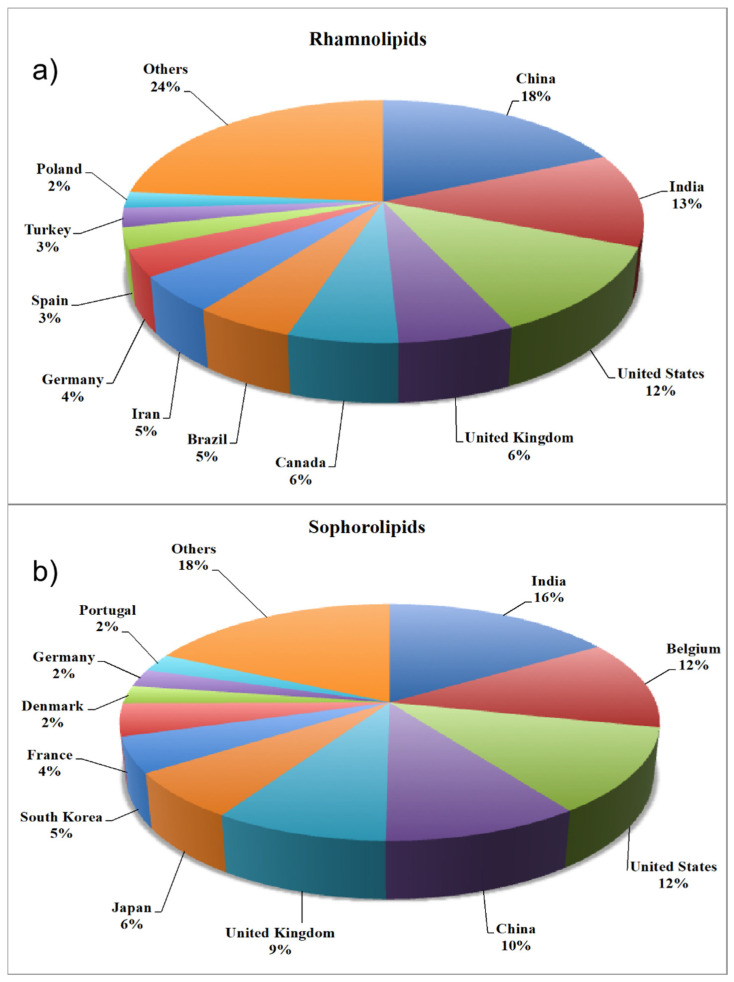
Distribution of published works regarding (**a**) rhamnolipids and (**b**) sophorolipids, by country.

On the other hand, Figure 2 shows the distribution of publications about rhamnolipids (Figure 2a) and sophorolipids (Figure 2b) by country. Surprisingly, Figure 2 is not in consonance with Figure 1, where the USA was the dominant country in terms of microbial biosurfactant production. Regarding microbial biosurfactant research, China and India currently lead the ranking, accounting for 18% and 16% of total published studies to date on rhamnolipids and sophorolipids, respectively.

From these two types of microbial biosurfactants, sophorolipids are only included in the CosIng database as surfactants, whereas rhamnolipids are included because of their emollient, emulsifier and skin conditioning properties. The sophorolipids included in the CosIng database are mostly produced by *Candida bombicola* and *Starmerella bombicola*. Concerning the use of sophorolipids contemplated in the CosIng, they can exert cleansing, antimicrobial, emulsifier, deodorant, anti-seborrheic, antioxidant, skin conditioning and skin-protecting capacities.

As an example, Hasani-Zadeh et al. [69] have detected the production of a new glycolipid microbial biosurfactant by *Mucor circinelloides*, which is able to reduce the surface tension of water to 26 mN/m. Moreover, it presents a huge emulsifying capacity for synthetic oils, such as engine oils. Nevertheless, this capacity is lowered in water emulsions systems containing natural oils like olive oil, although it is still higher than the emulsifying activity of Tween 80. Conversely, Kaur et al. [70] have reported the production of sophorolipids from food waste achieving a concentration of 115.2 g/L, after 92 h of fermentation, with overall volumetric productivity of 1.25 g/L·h.

Regarding their surfactant properties, they are able to reduce the surface tension of water by more than 30 mN/m and possess a CMC below 100 mg/L [14].

Other types of glycolipid microbial biosurfactants used in the cosmetic field are the mannosylerythritol lipids (MELs), which are extensively produced by different basidiomycetous yeasts like *Pseudozyma* [71]. Cosmetic applications on skincare (e.g., moisturization of dry skin), hair care (e.g., repair of damaged hair) as well as activation of fibroblast and papilla cells and antioxidant and protective effects in skin cells have been found [72].

### 4.2. Lipopeptides and Lipoproteins

These microbial biosurfactants, together with glycolipids, are the most studied in the literature [18,73]. They have a low CMC and can reduce the surface tension of water by more than 30 mN/m. They are mainly produced by *Bacillus subtilis*. Depending on the length of the fatty acid chain, their molecular weight can vary from 879 to 1620 Da, although most lipopeptide microbial biosurfactants possess a molecular weight of around 1000 Da [74,75,76,77]. Table 1 includes the molecular formula and molecular weight of different lipopeptides, the producer microorganism and their CMC (lower than 54 mg/L).

The most studied lipopeptide is surfactin produced by *B. subtilis* [83]. This microbial biosurfactant presents poor solubility in water, only being soluble at high pH (8–8.5), which can be a drawback to inclusion in cosmetic or pharmaceutical formulations. It can, however, be easily dissolved in organic solvents such as ethanol, methanol, butanol, chloroform, and dichloromethane [84]. Other microorganisms that produce lipopeptides with surfactant capacity are the genus Pseudomonas. As an example, *Pseudomonas libanensis* produces a lipopeptide microbial biosurfactant named viscosin [82].

From the above-mentioned microbial biosurfactants, only surfactin (as sodium surfactin) is included in the CosIng database as a cosmetic ingredient.

Lipopeptide microbial biosurfactants are widely used in cosmetics due to their anti-wrinkle and moisturizing properties and cleansing activity. Furthermore, they could be a potentially topical dermatological product, because of their good skin compatibility with low irritation [85].

### 4.3. Glycolipopeptides and Glycopeptides

**Figure 3:**
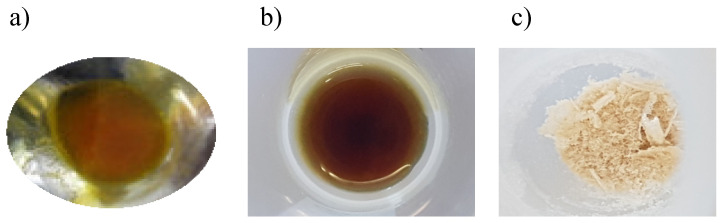
Macroscopic appearance of (**a**) lipopeptide, (**b**) rhamnolipid and (**c**) glycolipopeptide microbial biosurfactants.

These are the least known microbial biosurfactants, mainly produced by lactic acid bacteria [86,87,88]. These microbial biosurfactants present a different aspect in comparison to lipopeptides and glycolipids. In this sense, Figure 3 shows a picture of a lipopeptide and a glycolipid (rhamnolipid), in comparison to a glycolipopeptide produced by *Lactobacillus pentosus* [89].

As can be observed, lipopeptides and glycolipids are more yellowish and viscous than glycolipopeptides, possessing poor solubility in water. This appearance is observed for microbial lipopeptide and glycolipopeptide biosurfactants from corn steep liquor stream and lactic acid bacteria, respectively under specific extraction conditions [89]; but their appearance could change if other conditions are used. For their inclusion in aqueous formulations, they need to be heated or dissolved together with the oil phase. Contrarily, glycolipopeptides possess high solubility in water [90]. In fact, formulations containing up to 10 g/L of soluble glycolipopeptides have been found in the literature [91]. Macroscopically, glycolipopeptides are a white powder that is easy to handle and store. Regarding their physicochemical characterization, they have the capacity to form foam and are able to reduce the surface tension of water by between 16–27 mN/m with a CMC between 1 and 6 g/L [35,92,93,94], which is higher than that observed in glycolipids and lipopeptide microbial biosurfactants.

The most studied glycolipopeptide and glycopeptide microbial biosurfactants are those produced by lactic acid bacteria such as *Lactobacillus agilis* [33], *Lactobacillus plantarum* [93], *Lactobacillus paracasei* [35,95,96] and *L. pentosus* [32,34,89,96,97,98]. The concentration of lipids in the microbial biosurfactant extract can vary depending on the carbon source and fermentation conditions [35]. For instance, when glucose is used as a carbon source *L. paracasei* produces a glycolipopeptide, whereas when lactose is used as a carbon source the same strain produces glycopeptides or glycoproteins; showing that the nature of the carbon source plays an important role in the polymeric composition of microbial biosurfactants. The same fact was observed by other authors with microbial biosurfactants of different types like rhamnolipids produced by *Pseudomonas aeruginosa* [99] or lipopeptides produced by *Bacillus* strains [100].

Cyclopeptide and glycopeptide microbial biosurfactants are linked to the cell membrane of *Lactobacillus* and can be extracted using saline buffer phosphate or just buffer phosphate [34,96,97,101].

Some glycopeptide and glycolipopeptide microbial biosurfactants have shown antimicrobial activity against skin pathogens [96]. Therefore, at concentrations of 50 g/L, the glycolipopeptide produced by *L. pentosus* showed an important antimicrobial activity against *P. aeruginosa*, *Streptococcus agalactiae*, *Staphylococcus aureus*, *Escherichia coli*, *Streptococcus pyogenes* and *Candida albicans* similar to the antimicrobial activity shown by *L. paracasei*. Additionally, microbial biosurfactants produced by both microorganisms also showed significant anti-adhesive properties against all the microorganisms named above, except for *E. coli* and *C. albicans* [96]. Based on these studies, glycolipopeptides produced by *Lactobacillus* strains could be introduced in the cosmetic and personal care industry not only as a detergent but also as prebiotic ingredients as they are produced by probiotic bacteria and inhibit the growth of harmful skin microorganisms [14,96].

### 4.4. Bioemulsifiers with Surfactant Capacity

#### 4.4.1. Phospholipids

These substances are included in this classification owing to their origin as they can be obtained from vegetable or animal cells. Phospholipids are mostly classified as zwitterionic substances and are polar lipids. The phospholipids can be classified into two groups: glycerophospholipids and sphingolipids. Glycerophospholipids are composed of a glycerol base structure with two fatty acids esterified at the sn-1 and sn-2 positions, and a phosphorylated alcohol (choline, ethanolamine, inositol, or serine), obtaining phosphatidylcholine, phosphatidylethanolamine, phosphatidylinositol and phosphatidylserine derivates. Otherwise, sphingolipids are formed by the aliphatic amino alcohol sphingosine base structure, fatty acids and sugars or alcohols or phosphoric acid; and they can include sphingomyelin, gangliosides and cerebrosides [102,103].

The CosIng ingredient list includes various substances within the phospholipids category such as some glycine soy lipids or an alcohol soluble extract consisting predominantly of phospholipids. Other examples are sterols and triglycerides and hydrolysed phospholipids, obtained from different vegetable sources and hydrolysed with acids or enzymes [104].

It is interesting to remark that although phospholipids possess surfactant capacity, they are not included in the CosIng database as surfactants. They are used in hair and skin conditioning formulations [105], whereas in the food industry phospholipids are used as emulsifiers [106]. From a macroscopic point of view, phospholipids possess an appearance similar to glycolipids and lipopeptides.

#### 4.4.2. Polymeric Biosurfactants

The most studied polymeric microbial biosurfactants are polymeric emulsifiers with surfactant capacity produced by microorganisms. Of these, in turn, the most studied is emulsan, which is a polyanionic amphipathic heteropolysaccharide produced by *Acinetobacter calcoaceticus* [107]. The capacity of emulsan to reduce the surface tension of water is not great but it possesses a good emulsifier activity [10]. Other bioemulsifiers with surfactant activity are biodispersan and liposan [108]. In this regard, liposan is a water-soluble emulsifier with surfactant capacity and it is extracellularly produced by *Candida lipolytica*, composed of 83% carbohydrates and 17% proteins. It has been applied in the food and cosmetic industries as an emulsifier [109].

#### 4.4.3. Particulate Biosurfactants

The most studied particulate microbial biosurfactants are articulated vesicles, which are composed of proteins, phospholipids and polysaccharides [108]. These particulate microbial biosurfactants include phosphatidyl ethanolamine-rich vesicles, produced by *Acinetobacter* spp. [109]. Despite the fact that they can be considered microbial biosurfactants, their main role is as emulsifiers [10].

## 5. Comparison of Microbial Biosurfactants with Synthetic and Bio-Based Surfactants in the Cosmetic Industry

Due to the huge operational costs to obtain microbial biosurfactants compared to synthetic or bio-based surfactants [3], their uses have to be limited to the personal care and pharmaceutical industries, with the exception of those obtained directly from fermented agro-industrial streams, with a reduced cost, like the case of the microbial biosurfactants extracted from corn steep water [110,111]. This type of microbial biosurfactant extract is directly obtained from corn wet-milling waste streams, thus its production costs are as competitive as the synthetic ones. In fact, this finding was internationally patented, since the corn steep liquor stream involves a new source of microbial biosurfactants [112]. Recently, López-Prieto et al. [113] isolated and characterized the microorganism responsible for the production of microbial biosurfactants in this corn residue, noticing that it is a *Bacillus* strain with the capacity to generate both extracellular and cell-bound microbial biosurfactants. On the other hand, this microbial biosurfactant has already been tested in hair care [21,114,115] as well as skincare [116,117,118,119] formulations.

Microbial biosurfactants were applied to cosmetic, personal care and pharmaceutical formulations, achieving interesting results with fewer side effects than those produced by synthetic surfactants. Table 2 summarizes some studies that show different applications of microbial biosurfactants among these fields.

Table 2 shows that microbial biosurfactants have a wide variety of applications in the cosmetic and personal care industry, acting as anti-ageing agents or cleansers, as well as in the pharmaceutical field, where they can be applied to different products owing to their antimicrobial capacity, which means they are regarded as substances with huge potential.

Among all microbial biosurfactants used in the cosmetic industry, lipopeptides and glycolipids are the ones selected due to their multifunctional profile, especially based on their physiochemical properties and biological activities [85,140].

A notable example for the cosmetic industry is the development of new formulations with sunscreen properties. Rincón-Fontán et al. [116] designed and characterized a greener sunscreen formulation based on mica powder (a mining silicate mineral) and a lipopeptide microbial biosurfactant extract from corn steep liquor. A synergistic effect was observed between them in relation to the protection provided against the harmful effects of the sun. In addition, the same authors in another work [117] tested the same compounds (mica and microbial biosurfactant extracts) to stabilize Pickering emulsions containing vitamin E using a triangular design. It was detected that the presence of this microbial biosurfactant extract improved the emulsion volume (EV) up to 70% after 22 days.

Ferreira et al. [91] used a glycolipopeptide microbial biosurfactant obtained from *L. paracasei* as a stabilizing agent in oil-in-water (O/W) emulsions containing essential oils and a natural antioxidant extract from grape seeds. These formulations were evaluated using the sulforhodamine B (SRB) assay (with mouse fibroblast cell line 3T3) and compared with SDS. The results showed that O/W emulsions containing 10 g/L of microbial biosurfactant and 5 g/L of the antioxidant extract showed EV values about 100% after 7 days of emulsion formation. On the other hand, formulations containing 5 g/L of microbial biosurfactant presented cell proliferation values of 97%, whereas 0.5 g/L of SDS showed a strong inhibitory effect.

Other interesting applications related to personal care applications were found. Das et al. [141] used a microbial biosurfactant, obtained from *Nocardiopsis* VITSISB, in a toothpaste formulation, replacing SLS, which is normally used in commercial toothpaste as a surfactant. The results indicated that microbial biosurfactant is more efficient and less toxic than chemical surfactant. Farias et al. [142] formulated several types of mouthwash with a microbial biosurfactant, chitosan (from fungus biomass) and peppermint (*Mentha piperita*) essential oil. Additionally, three microbial biosurfactants obtained from *Pseudomonas aeruginosa* UCP 0992 (PB), *Bacillus cereus* UCP 1615 (BB) and *Candida bombicola* URM 3718 (CB) were examined. The antimicrobial action against oral microorganisms and toxicity using the MTT (3-(4,5-dimethylthiazole-2-il)-2,5-diphenyltetrazolium bromide)) method for the L929 (mouse fibroblast) and RAW264.7 (mouse macrophage) cell lines of the mouthwashes were evaluated. The results showed that the mouthwashes, containing the microbial biosurfactant and other natural products, represented lower toxicity than the commercial ones.

Nevertheless, these studies did not take into account the biodegradation of these surface-active compounds in cosmetic formulations. This is a challenge, since immediate microbial biosurfactant biodegradation would not be desirable before realizing their role in the cosmetic formulation. For that reason, Rodríguez-López et al. [143] recently studied the biodegradability of a lipopeptide microbial biosurfactant, from corn steep liquor, under different environmental conditions, without the addition of external microbial biomass. They found that the t_1/2_ of this microbial biosurfactant (time to achieve 50% of biodegradation) was no lower than 35 days at pH 5.

Conversely, there are several works that have demonstrated the toxicity of surfactants and bio-based surfactants, which can reach animals through feeding or through the skin. When the surfactant concentration in water is too high, surfactants can enter the gills, blood, kidney, pancreas, gallbladder and liver and produce an aquatic toxicity effect [144]. Surfactants can also produce allergic reactions on the skin when they are included in cosmetic formulations. Table 3 summarizes some examples of side effects produced by surfactants. For instance, the bio-based surfactant lauryl glucoside, a non-ionic biodegradable surfactant obtained from plants, is most frequently involved in allergies related to sunscreen formulations [145].

The side effects of surfactants can be avoided by using bio-based surfactants or microbial biosurfactants. However, the challenge to incorporate ingredients of natural origin or microbial biosurfactants in cosmetic products is the safety assessment according to the Scientific Committee on Consumer Safety [156]. For that reason, in vitro tests are used to evaluate the safety of cosmetic ingredients due to the prohibition of animal tests in Europe in 2004 and in 2009 for cosmetics and for cosmetic ingredients, respectively [157,158].

Kim et al. [159] tested the toxicity of MEL microbial biosurfactant from *Candida* sp. SY16 in mouse fibroblast L929 cells after 48 h of exposure using a Neutral Red (NR) assay. The midpoint toxicity value (NR_50_) of MEL was higher (5 g/L) in comparison with synthetic surfactants such as SDS and LAS (linear alkylbenzene sulfonate), which had NR_50_ values of 0.05 g/L and 0.01 g/L, respectively. The data clearly suggest that MEL-SY16 is not harmful to human skin and eyes when compared to synthetic surfactants.

Morita et al. [160] assessed the cell viability of SDS-damaged human skin cells recovered by the addition of MEL microbial biosurfactant (e.g., MEL-A), having a recovery rate of 73% and 91% using MEL-A solutions of 5 wt% and 10 wt%, respectively. This makes it a novel and cost-effective moisturizer agent. A similar study was carried out by Yamamoto et al. [161] but using MEL derivates (e.g., MEL-A, MEL-B and MEL-C). The cell viability rates were over 80% with most of the MEL tested; in addition, the stratum corneum water content in the skin was increased when an aqueous solution of MEL-B (5 wt%) was used.

Burgos-Díaz and collaborators [162] evaluated the cytotoxicity effects of a microbial biosurfactant produced by *Sphingobacterium detergens* (it was fractionated in two portions: fraction A corresponding to a mixture of phospholipids; and fraction B consisting of a polar lipid mixture) in 3T3 fibroblast and HaCaT keratinocyte cell lines after 24 h of exposure using the Neutral Red Uptake (NRU) and MTT assays. It was observed that both fractions showed similar cytotoxicity in fibroblasts and keratinocytes cell lines; however, it was worth noting that fraction B showed lower cytotoxicity than those obtained using SDS, indicating low skin irritability.

Rodríguez-López et al. [98] have studied the irritant effect of microbial biosurfactants in comparison to SDS at the same concentration (1 g/L), observing that SDS produced the lysis of the small vessel in chorionallantoic membrane (CAM) of the hen’s egg, whereas no side effects were observed by the glycolipopeptide microbial biosurfactant produced by *L. pentosus* or by the microbial biosurfactant extract composed of lipopeptides obtained from a residual stream of the corn milling industry (corn steep liquor), spontaneously fermented by *Bacillus* strains.

On the other hand, it is important to remark that although chemical surfactants possess a low CMC, they are used in high concentrations to be effective in cosmetic and pharmaceutical formulations. Some of these formulations contain up to 30% of surfactants. Table 4 includes some cosmetic and pharmaceutical formulations and their surfactant content.

According to Table 4, large amounts of surfactants are contained in cosmetic and personal care formulations, achieving in some cases values up to 50%. As for the side effects that surfactants can produce, it is necessary to reduce the quantity of surfactants in formulations while trying to keep the same efficiency. If this is not possible, microbial biosurfactants could be the best candidates to substitute synthetic surfactants in marketed products, as they avoid the side effects produced by synthetic ones and are more environmentally friendly [167]. Contrarily to chemical and bio-based surfactants, microbial biosurfactants are easily biodegraded by microorganisms [143,168,169] and they should not be cytotoxic as they are composed of lipids, sugars and proteins, making them a suitable ingredient for food, cosmetics and pharmaceuticals formulations [10,14,15]. The use of microbial biosurfactants in these industrial sectors would decrease the number of cases of allergies and side effects produced by surfactants included in cosmetic formulations [145,146,170]. This statement is corroborated by the higher lethal concentration (LC_50_) and effective concentration (EC_50_) of microbial biosurfactants in comparison to synthetic surfactants [171].

## 6. Concluding Remarks and Future Perspectives

The increasing demand for surfactants by the cosmetic and personal care and pharmaceutical industries has generated huge consumption of petroleum-based synthetic surfactants, which are often toxic, irritant and non-biodegradable. Bio-based surfactants have come along as an alternative to synthetic surfactants, obtained from the petrochemical industry. Bio-based surfactants are compounds obtained using renewable sources and biomass. Hence, these kinds of surface-active compounds prevent the use of petrochemical sources; however, although vegetable and animal oils are used in their production, they are obtained through a chemical reaction that involves the consumption also of non-renewable sources. Nevertheless, microbial biosurfactants, which are produced by microorganisms, using biological reactions, as secondary metabolites, could represent a promising alternative since they are composed of lipids, carbohydrates or proteins, which makes them more biocompatible and biodegradable than their synthetic and bio-based counterparts. Additionally, they present low toxicity, stability in extreme conditions and several bioactivities.

Therefore, if microbial biosurfactants are less toxic and more biodegradable than chemical surfactants, what is wrong with microbial biosurfactants? Why are they not being included in cosmetic, personal care and pharmaceutical formulations? The main problem is related to the biotechnological production of microbial biosurfactants, which means a higher production cost, as they are secondary metabolites. Moreover, the biotechnological production of microbial biosurfactants involves an important cost regarding not only the nutritional medium but also the extraction and purification steps. Therefore, it is necessary to seek an increase in the overall productivity of microbial biosurfactants by obtaining higher producer microorganisms and by exploring the use of cost-competitive nutritional media, including the use of fermented residual streams (like corn steep liquor) where microbial biosurfactants can be produced spontaneously. Lastly, due to the current trend towards green consumption, it is expected there will be a significant effort to develop cosmetic, personal care and pharmaceutical formulations in which synthetic surfactants are replaced by renewable and environmentally friendly microbial biosurfactants. Therefore, international cosmetic regulations are required to incorporate microbial biosurfactants as a separate group of surfactants in order to obtain more biocompatible and greener formulations.

## Figures and Tables

**Table 1:** Lipopeptide microbial biosurfactants. Microbial sources, molecular formula and weight and critical micellar concentration (CMC).

Microbial Biosurfactant	Producer Microorganism	Molecular Formula	Molecular Weight (Da)	CMC (mol/L)	Ref.
Surfactin	*Bacillus subtilis*	C_53_H_93_N_7_O_13_	1036.8	9.4 × 10^−6^	[78]
Iturin A	*Bacillus subtilis*	C_48_H_74_N_12_O_14_	1043.2	2.5 × 10^−7^	[79]
Fengycin	*Bacillus subtilis*	C_72_H_110_N_12_O_20_	1463.7	1.2 × 10^−6^	[80]
Lichenysin C	*Bacillus licheniformis*	C_52_H_91_N_8_O_12_	1020.7	1.5 × 10^−5^	[81]
Viscosin	*Pseudomonas libaniensis*	C_54_H_95_N_9_O_16_	1126.4	4.8 × 10^−5^	[82]

**Table 2:** Uses of microbial biosurfactants among cosmetic, personal care and pharmaceutical industries.

Type of Microbial Biosurfactant	Application	Ref.
Rhamnolipid	Anti-ageing product	[120]
	Cleanser in shampoos	[121]
	Anti-adhesive activity	[122]
Rhamnolipid/Sophorolipid	Cleanser for antidandruff shampoo Moisturizing skin cleanser Body cleanser	[123]
Sophorolipid	Cleanser in shower gel and shampoo	[124]
	Body washer	[125]
	Anti-inflammatory agent	[126]
Glycolipid	Cleanser in shampoo formulation	[127]
	Antifungal activity	[128]
	Hair-care conditioning polymers	[129]
Lipopeptide	Hair care formulation	[21]
	Rosemary oil/water emulsions	[89]
	Dyed hair care formulation	[114]
	Stabilizing agent for antidandruff formulations based on Zn pyrithione powder	[115]
	Sunscreen formulations based on mica powder	[116]
	Pickering emulsions containing Vitamin E	[117]
	Stabilizing agent of vitamin C	[118]
	Antiacne formulation	[119]
	Antiviral agent	[130]
	Antimicrobial agent in silver plasmonic nanoparticles	[131]
	Nanoemulsions and nanocrystals for dermal application	[132]
	Permeation of pharmaceutical compounds by silicone membranes	[133]
	Antimicrobial agent	[134]
Glycolipopeptide	Rosemary oil/water emulsions	[34,35]
	Cosmetic formulation with antioxidants	[91]
	Bioactivity against skin pathogens (antimicrobial and anti-adhesive agent)	[96]
Glycolipopeptide/Lipopeptide	Rosemary oil/water emulsions	[89]
	Preservative and irritant agent	[98]
MELs	Anti-ageing product	[135]
	Prevent skin roughness	[136]
	Makeup product	[137]
	Antimicrobial agent	[138]
Oligomeric biosurfactant	Conditioning agent for hair products	[139]

**Table 3:** Side effects related to synthetic and bio-based surfactants present in cosmetic and personal care formulations.

Surfactant	CAS Number	Category	Toxicity	Formulation	Ref.
Lauryl glucoside	110615-47-9	Bio-based	Skin reaction	Sunscreens	[145]
C12-14 hydroxyalkyl hydroxyethyl sarcosine	---	Synthetic	Allergic contact dermatitis	Ampholytic surfactants in soaps and shampoos	[146]
Capryloyl glycine	14246-53-8	Not defined	Skin reaction	Polyfunctional ingredient in cosmetic leave-on and rinse-off products	[147]
Octoxynol-9	9002-93-1	Synthetic	Scarification	Shampoo and body washes	[148]
Benzalkonium chloride	63449-41-2	Synthetic	Eye irritancy	Cationic surfactant employed in rash crème, foot odour powder, facial lotion, cleanser, among others	[149]
Quaternium-15	4080-31-3/51229-78-8	Synthetic	Skin allergy and irritant contact dermatitis	Skin cleanser	[150]
Cocamidopropyl betaine	61789-40-0	Bio-based	Skin allergy and irritant contact dermatitis	Skin cleanser	
Sodium lauryl ether sulfate	68585-34-2	Synthetic	Erythema and skin scaling	Shampoos and shower gels	[151]
Sodium lauroyl glutamate	29923-31-7	Bio-based	Transepidermal water loss	Lotions, creams, shampoos	[152]
Acetyl tyrosinamide	1948-71-6	Not defined	Erythema and edema postapplication	Gel and skin plumping cream formulation	[153]
Coco-betaine	68424-94-2	Synthetic	Eczematous lesions	Shampoos	[154]
Laureth-11 carboxylic acid	27306-90-7	Not defined	Eye irritancy	Bath and shower products	[155]
Palmitamidopropyltrimonium chloride	51277-96-4	Not defined	Eye irritancy	Hair conditioner	
Linoleamidopropyl PG-dimonium chloride phosphate	243662-49-9	Not defined	Eye irritancy	Bar soap, baby shampoo, After shave	

**Table 4:** Surfactant content in cosmetic, personal care and pharmaceutical products.

Formulation Type	Surfactant	CAS Number	Content (%)	Ref.
Clear liquid shampoo	Triethanolamine lauryl sulfate	139-96-8	50	[163]
Liquid cream shampoo	Triethanolamine lauryl sulfate	139-96-8	35	
Cream shampoo	Sodium lauryl sulfate	151-21-3	38	
Gel shampoo	Triethanolamine lauryl sulfate	139-96-8	28	
Foaming-type cream	Monoethanolamine lauryl sulfate + ethylene glycol monostearate	4722-98-9 + 111-60-4	11	
Hair colorant	Stearic acid + triethanolamine + glyceryl mono stearate	57-11-4 + 102-71-6 + 31566-31-1	28	
Colour shampoos	Ammonium lauryl alcohol sulfate	2235-54-3	30	
Powder shampoo	Sodium lauryl sulfate	151-21-3	20	[163,164]
Liquid hand soap	Sodium laureth sulfate + coco-betaine + lanolin + cocamide diethanolamine	3088-31-1 + 68424-94-2 + 8006-54-0 + 68603-42-9	24	[165]
Cleansing gel	Sodium laureth sulfate + cocamidopropyl betaine	3088-31-1 + 61789-40-0	19	
Shower scrub	Sodium laureth sulfate + sodium cocoyl glutamate + cocamidopropyl betaine	3088-31-1 + 68187-30-4 + 61789-40-0	41	[166]
Shower gel	Sodium coco-sulfate + coco glucoside + glycerol oleate	97375-27-4 + 110615-47-9 + 25496-72-4	14	

## Data Availability

Not applicable.
